# Predictors of Psychiatric Boarding in the Emergency Department

**DOI:** 10.5811/westjem.2014.10.23011

**Published:** 2014-11-26

**Authors:** Ryan K. Misek, Ashley E. DeBarba, April Brill

**Affiliations:** Midwestern University, Chicago College of Osteopathic Medicine, Department of Emergency Medicine, Downers Grove, Illinois

## Abstract

**Introduction:**

The emergency psychiatric care is system is overburdened in the United States. Patients experiencing psychiatric emergencies often require resources not available at the initial treating facility and frequently require transfer to an appropriate psychiatric facility. Boarding of psychiatric patients, defined as a length of stay greater than four hours after medical clearance, is ubiquitous throughout emergency departments (EDs) nationwide. Boarding is recognized as a major cause of ambulance diversions and ED crowding and has a significant adverse impact on healthcare providers, patient satisfaction, and hospital costs. We sought to identify differences between patients who boarded versus patients who did not board, to identify factors amenable to change and identify interventions that could lead to a decrease in overall psychiatric patient length of stay and improve patient care.

**Methods:**

This study is a retrospective multicenter cohort study of all patients assessed to require inpatient psychiatric hospitalization at two community EDs in Illinois from July 1, 2010 through June 30, 2012. We identified 671 patients and collected insurance status, sex, age, time of arrival, time of disposition and time of transfer.

**Results:**

There was a statistically significant difference in the insurance status between the cohort of patients boarding in the ED compared to non-boarders prior to inpatient psychiatric admission. Our study identified 95.4% of uninsured patients who were boarded in the ED, compared to 71.8% of Medicare/Medicaid patients and 78.3% of patients with private insurance (χ^2^=50.6, df=2, p<0.001). We found the length of stay to be longer for patients transferred to publicly funded psychiatric facilities compared to those transferred to private facilities, with a mean time spent in the ED of 1,661 minutes and 705 minutes, respectively (p<0.001). Patients with Medicare/Medicaid were nearly twice as likely to return to the ED for psychiatric emergencies than self-pay and privately insured patients, requiring repeat inpatient psychiatric admission (estimate=0.649, p=0.035, OR=1.914).

**Conclusion:**

This study found that unfunded patients boarded significantly longer than Medicare/Medicaid and privately insured patients. Patients with private insurance boarded longer than those with Medicare/Medicaid. Patients transferred to publicly funded facilities had significantly longer ED length of stay than patients transferred to private facilities.

## INTRODUCTION

Emergency department (ED) “crowding” is an international problem that has received much attention as it is associated with increased morbidity and mortality.^3^ A major contributing factor in ED crowding is patient boarding. A 2008 American College of Emergency Physicians survey found that nearly 80% of EDs reported boarding psychiatric patients who were not admitted to their facility.^1^ Psychiatric patients have been “deinstitutionalized” over recent decades with their care shifting from the inpatient to outpatient setting. In 1970, state and county psychiatric inpatient beds numbered approximately 400,000 and in 2006 numbered at just 50,000.^4^,^5^ Concurrently, patients with substance abuse or mental health problems have surged from 5.4% of all ED visits in 2000 to 12.5% of the 95 million ED visits in 2007.^6^ The decrease in inpatient psychiatric beds combined with the increase in mental health-related ED visits have amplified the number of patients boarding in the ED.^1^

Recently, Chang et al.^7^ explored characteristics of patients who boarded over 24 hours at five Massachusetts’ hospitals and found that homelessness, inter-hospital transfer, public insurance and use of restraints or sitters placed the patient at a higher risk for boarding in the ED.^7^ Only 2.6% of Massachusetts residents were uninsured in the state’s integrated healthcare system at the time of the study. A similar study by Weiss et al.^8^ found that a medical need for hospitalization, restraint use and use of diagnostic imaging as factors associated with prolonged boarding.^8^ Additional research identified high variability in boarding times between hospitals and postulated that this was due to differences in psychiatric services offered and inpatient bed availability.^9^ In pediatric patients requiring inpatient psychiatric admission, one study found patients who boarded were more likely to have presented over the weekend, during school vacation or with suicidal ideations.^10^ A prospective cohort study in which psychiatric clinicians were surveyed found that staff and bed availability, need for clinical stability, obtaining additional information and patients’ financial issues as the rate limiting step for discharge of psychiatric patients.^9^ While several studies have evaluated the characteristics of psychiatric boarders in Massachusetts, few have studied this cohort of psychiatric boarders in a state without an integrated healthcare system in place.^11^,^12^ Our hypothesis was that patients without insurance are at highest risk for boarding in the ED. Through the identification of predictive factors that lead to ED boarding we hope to bring attention to the need for increased psychiatric services for this group as well as improve ED efficiency and allocation of resources.

## METHODS

The authors conducted a retrospective multicenter cohort study of adult patients presenting to two suburban teaching hospital EDs in Illinois from July 1, 2010 through June 30, 2012 assessed to require inpatient psychiatric hospitalization. Exclusion criteria consisted of patients under 18 years of age, patients over 65 years of age, patients who required stabilization of a medical condition prior to transfer, patients who were pregnant, and patients who were discharged from the ED prior to transfer to a psychiatric facility. The main outcome was placement into a psychiatric facility or boarding in the ED. All patients were evaluated in the ED and deemed to require inpatient psychiatric treatment by the attending emergency physician. Institutional review board approval was granted by the host hospital system and the host educational institution.

The patients determined to require inpatient psychiatric hospitalization were transferred to one of approximately 36 regional psychiatric facilities, as neither study hospital had an in-house psychiatric unit at the time of the study. All decisions regarding patient disposition were approved by an attending physician. Placement at psychiatric facilities was facilitated through a regional psychiatric coordination service that assists with patient placement to appropriate facilities after disposition decision is made by the ED attending physician.

The hospital’s medical record department generated a list of patients meeting inclusion criteria into an Excel spreadsheet. Using patient encounter numbers, data were collected from each chart in the hospital’s general medical record system and entered into the spreadsheet by student research assistants. One of the researchers (RM) then coded these data. De-identified coded data were sent to the host institution statistician for analysis. We analyzed data using statistical package for the social sciences/predictive analytics software statistics.

The main outcome measure was placement into a psychiatric facility or boarding, defined as remaining in the ED for four hours or longer following disposition decision. Patients who boarded were followed to see if they re-presented to one of the two participating EDs within a year of initial presentation for a complaint requiring psychiatric evaluation. Study participants were evaluated for the following: time from presentation to the ED to decision to admit; time from admission decision to transfer to a psychiatric facility; time of ED presentation to transfer to a psychiatric facility; and date of first re-presentation, or “bounce back,” to the ED within 12 months following initial ED psychiatric visit. We collected additional demographic and treatment data to compare the boarding to the non-boarding cohort to examine factors that may contribute to the “bounce back.” These factors included race, sex, age and insurance coverage (private, Medicare/Medicaid or self-pay).

We conducted chi-square analyses to compare insurance status between cohorts of ED boarders and nonboarders. An analysis of variance test was used to analyze differences in time to disposition and time from disposition to transfer between the different insurance status groups. Secondary outcomes investigated insurance status and the likelihood of “bounce back.” To investigate this, we used generalized linear models specifying a binomial distribution and logit link function to test the importance of the predictor variables. Models 1 and 2 only contained a single predictor variable, insurance status and boarder status, respectively. Model 3 contained the two predictor variables of interest, as well as three variables - sex, race/ethnicity, and age - as covariates.

## RESULTS

Overall, 910 patients met inclusion criteria of which 671 qualified for the study after exclusion criteria. Of these, 81.4% of the 671 patients were identified in the ED boarder cohort. Uninsured patients were boarded in the ED 95.4% before psychiatric inpatient admission (χ^2^=50.6, df=2, p<0.001) compared to 71.8% of Medicare/Medicaid patients (χ^2^=50.6, df=2, p<0.001) and 78.3% of patients with private insurance (χ^2^=50.6, df=2, p<0.001) (Table 1). Mean ED length of stay was 705 minutes for patients transferred to private psychiatric facilities compared to 1661 minutes when transferred to public psychiatric facilities (p<0.001).

No significant differences were found in time from ED arrival to decision to admit for patients regardless of insurance status. Time from ED arrival to decision to admit for privately insured, Medicare/Medicaid, and uninsured patients had a mean time of 241 minutes, 216 minutes, 226 minutes, respectively, before transfer to a private psychiatric facility. Time from ED arrival to decision to admit for privately insured, Medicare/Medicaid, and uninsured patients had a mean time of 279 minutes, 228 minutes, and 360 minutes, respectively, before transfer to a public psychiatric facility.

Secondary outcomes investigated the correlation of insurance status and boarding in the ED on the likelihood of “bounce back,” defined as the return to the ED at any point during the study period requiring inpatient psychiatric admission. For Model 1, we found a significant effect of insurance status on “bounce back” (estimate=0.649, p=0.035). Specifically, patients with Medicare/Medicaid were nearly two times as likely to be associated with bounce back (OR=1.914). In contrast, being a boarder was not significantly associated with “bounce back” (p=0.405, OR=0.809). Using the multivariate generalized linear model produced similar results to our chi-square analysis. Only insurance status was significantly related to “bounce back” with Medicaid/Medicare coverage significantly related to “bounce back” (estimate=0.652, p=0.037, OR=1.919). Being a boarder, sex, race/ethnicity, nor age were related to “bounce back.”

## DISCUSSION

ED crowding is a multifactorial problem faced by hospitals nationwide. The boarding of patients is recognized as a major cause of ambulance diversions and ED crowding, and has a significant impact on healthcare providers, patient satisfaction, and hospital costs.^2^,^13^ Psychiatric boarding is becoming an increasingly more prevalent practice disrupting patient care as well as ED throughput and is only expected to increase with the continued closures of state-run psychiatric facilities. Our findings show that indeed patients without insurance are at highest risk for boarding in the ED. Medicare/Medicaid patients, in our study, were most likely to require repeat inpatient psychiatric admission. This could be because Medicare/Medicaid patients are better connected to the healthcare system than the uninsured; further research is needed to investigate this correlation. We have also identified at risk cohorts of psychiatric patients, uninsured and Medicare/Medicaid, for whom we can improve care and ED throughput.

There were no significant differences found between insurance status and the length of time it took from initial ED presentation to disposition by the emergency physician. This suggests that the emergency physician delivers care that identifies psychiatric patients requiring inpatient hospitalization in a consistent manner regardless of insurance status. This implies that the time for disposition by the emergency physician is not a confounding factor for increased boarding time of psychiatric patients in the ED and that the main variable affecting boarder versus non-boarder status is the insurance coverage of the patient.

Interestingly, our study found that privately insured patients were boarded more often than publicly insured patients (78.3% vs 71.8%, p<0.001). This could be due to differences in the approval process that is required prior to psychiatric facility placement of these patients. Privately insured patients often require authorization from their insurance company prior to being admitted at a psychiatric facility, whereas Medicare/Medicaid patients do not require pre-authorization. A small convenience sample of cases found an average wait time of 38 minutes for authorization, although 10% of these privately insured patients waited an hour or longer for approval.^14^

Research on psychiatric care in the ED is limited. Several prior studies analyzed characteristics of psychiatric patients in Massachusetts, where there were more psychiatric services compared to other parts of the country.^7^–^9^,^14^ Our study evaluated another region of the country, one in which psychiatric care is lacking after repeated cuts to inpatient and outpatient psychiatric services, and provides insight into challenges faced by community emergency providers.

One challenge faced by providers of psychiatric care in the U.S. has been the shrinking number of psychiatric beds available to inpatients, which has decreased by 14% nationally from 2005 to 2010. In 2005, there were 50,509 state psychiatric beds available, and by 2010, 43,318. Nationwide, closures reduced the number of beds available in the combined 50 states to 28% of the number considered necessary for minimally adequate inpatient psychiatric services.^15^

Secondary outcomes identified a population of psychiatric patients who were likely to return to the ED during the study period and require inpatient psychiatric admission. Medicare/Medicaid patients were nearly twice (OR=1.919) as likely to return to the ED during the 24-month study period requiring psychiatric admission. Ironically, these patients spent the least time boarding in the EDs compared to privately funded patients. The variables of age, sex, boarder status, race/ethnicity, were not found to be significantly related to “bounce back.” This suggests that Medicare/Medicaid patients may be a population at risk for psychiatric decompensation. Whether this is due to lack of outpatient options or other factors should be further investigated.

## LIMITATIONS

Limitations of this study, as with any retrospective cohort study, are an increased risk for errors due to confounding and selection bias. We attempted to limit selection bias as data were collected by research assistants blinded to the primary and secondary outcomes. Furthermore, data were analyzed by an outside statistician who was also blinded to the outcome measures. While it is difficult to establish a cause-and-effect relationship from retrospective cohort studies, our research identifies several areas for future prospective studies to include the relationship of insurance status to boarder status and frequency of return to the ED. Another limitation is that the study was limited to patients from two community EDs in Illinois, and results may not be applicable to other regions or hospital systems. Results on “bounce back” may also be skewed as data were not available on patients who may have presented to a hospital outside of our catchment area.

## CONCLUSION

Our study found patients without insurance boarded significantly longer than Medicare/Medicaid and privately insured patients. In addition, patients with private insurance boarded longer than those with Medicare/Medicaid. Secondary analysis identified significantly longer ED length of stay for patients transferred to publicly funded facilities compared to those transferred to private facilities.

Our research adds another perspective regarding characteristics of psychiatric boarders and which psychiatric patients decompensate following antecedent psychiatric admission. The boarding of psychiatric patients has been identified as a problem that affects EDs of all types across the U.S. and is familiar to most emergency physicians. Further research is needed to delineate which psychiatric patients board and “bounce back” so that future interventions can be made on their behalf to enhance their care, reduce their time spent boarding and prevent psychiatric decompensation. Significant benefits for the patient, hospital, and ED staff may be realized by improving the placement and management for patients requiring inpatient psychiatric treatment.

## Figures and Tables

**Table 1:** Insurance effect on psychiatric boarding in the emergency department.

		Boarder		
			
		Yes	No	Total
Insurance status				
Private	Count	94	26	120
	% within insurance status	78.3%	21.7%	100%
	% within boarder	17.2%	20.8%	17.9%
	% of total	14.0%	3.9%	17.9%
Medicare/medicaid	Count	224	88	312
	% within insurance status	71.8%	28.2%	100%
	% within boarder	41.0%	70.4%	46.5%
	% of total	33.4%	13.1%	46.5%
Self-pay	Count	228	11	239
	% within insurance status	95.4%	4.6%	100%
	% within boarder	41.8%	8.8%	35.6%
	% of total	34.0%	1.6%	35.6%
Total	Count	546	125	671
	% within insurance status	81.4%	18.6%	100%
	% within boarder	100%	100%	100%
	% of total	81.4%	18.6%	100%

## References

[1] ACEP (2008). Psychiatric and Substance Abuse Survey 2008.

[2] Falvo T, Grove L, Stachura R (2007). The opportunity loss of boarding admitted patients in the emergency department. Acad Emerg Med.

[3] Hoot R, Aronsky D (2008). Systematic review of emergency department crowding: causes, effects, and solutions. Ann Emerg Med.

[4] Tuttle GA Report of the Council on Medical Service, American Medical Association: access to psychiatric beds and impact on emergency medicine.

[5] Alakeson V, Pande N, Ludwig M (2010). A plan to reduce emergency room ‘boarding’ of psychiatric patients. Health Aff.

[6] Owens PL, Mutter R, Stocks C (2010). Mental Health and Substance Abuse-Related Emergency Department Visits among Adults, 2007. Healthcare Cost and Utilization Project (HCUP) Statistical Briefs.

[7] Chang G, Weiss AP, Orav EJ (2012). Bottlenecks in the Emergency Department: the psychiatric clinicians’ perspective. Gen Hosp Psychiatry.

[8] Weiss AP, Chang G, Rauch SL (2012). Patient and practice-related determinants of emergency department length of stay for patients with psychiatric illness. Ann Emerg Med.

[9] Chang G, Weiss AP, Orav EJ (2011). Hospital variability in emergency department length of stay for adult patients receiving psychiatric consultation: a prospective study. Ann Emerg Med.

[10] Wharff EA, Ginnis KB, Ross AM (2011). Predictors of psychiatric boarding in the pediatric emergency department: implications for emergency care. Pediatr Emerg Care.

[11] Stephens RJ, White SE, Cudnik M (2014). Factors Associated with Longer Length of Stay for Mental Health Emergency Department Patients. J Emerg Med.

[12] Pitts SR, Vaughns FL, Gautreau MA (2014). A cross-sectional study of emergency department boarding practices in the United States. Acad Emerg Med.

[13] Park JM, Park LT, Siefert CJ (2009). Factors associated with extended length of stay for patients presenting to an urban psychiatric emergency service: a case-control study. J Behav Health Serv Res.

[14] Funkenstein, Malowney M, Boyd JW (2013). Insurance Prior Authorization Approval Does Not Substantially Lengthen the Emergency Department Length of Stay for Patients With Psychiatric Conditions. Ann Emerg Med.

[15] Torrey EF, Entsminger K, Geller J (2008). The shortage of public hospital beds for mentally ill persons.

